# A simple way to identify non-viable cells within living plant tissue using confocal microscopy

**DOI:** 10.1186/1746-4811-4-15

**Published:** 2008-06-23

**Authors:** Elisabeth Truernit, Jim Haseloff

**Affiliations:** 1 University of Cambridge, Department of Plant Sciences, Downing Site, Cambridge, CB2 3EA, UK; 2 INRA, Centre de Versailles, Institut Jean-Pierre Bourgin, Laboratoire de Biologie Cellulaire, Route de St-Cyr, 78026 Versailles cedex, France

## Abstract

**Background:**

Plant cell death is a normal process during plant development. Mutant plants may exhibit misregulation of this process, which can lead to severe growth defects. Simple ways of visualising cell death in living plant tissues can aid the study of plant development and physiology.

**Results:**

Spectral variants of the fluorescent SYTOX dyes were tested for their usefulness for the detection of non-viable cells within plant embryos and roots using confocal laser-scanning microscopy. The dyes were selective for non-viable cells and showed very little background staining in living cells. Simultaneous detection of SYTOX dye and fluorescent protein (e.g. GFP) fluorescence was possible.

**Conclusion:**

The fluorescent SYTOX dyes are useful for an easy and quick first assay of plant cell viability in living plant samples using fluorescence and confocal laser-scanning microscopy.

## Background

Plant cell death is an integral part of plant growth and development and can also occur as a reaction to wounding or pathogen attack [1-3]. A well-known example of regulated cell death during normal plant development takes place during the maturation of xylem cells [4]. Plant cell death can also be seen during anther development [5], in lateral root cap cells at the end of the lateral root cap [6], and in many other tissue types [1].

Not surprisingly, mutations that lead to the premature death of certain plant cells have a detrimental effect on plant development. In the *Arabidopsis mosaic death1 *(*mod1*) mutant, for example, patches of cells within different organs die prematurely, leading to severe growth defects [7]. The *Arabidopsis tornado *(*trn*) mutant shows misspecification of some root epidermal cells as lateral root cap cells. These cells die in the elongation zone, as lateral root cap cells would normally do, and this results in the formation of gaps in the epidermis. As a consequence, *trn *roots are impaired in growth [8].

To study plant development, it is essential to have simple means to identify non-viable cells within living plant tissue. Although different modes of plant cell death exist in plants [9], one common characteristic of cell death is that the plasma membrane of dying cells ceases to function as a selective barrier. Hence, some dyes that cannot penetrate through the plasma membrane of living cells can be used to stain internal components of non-viable cells (= dye exclusion method). For example, trypan blue has been used to stain non-viable cells during pathogen-induced cell death (e.g. [7]). Non-viable cells were stained blue and could be observed with light microscopy. However, as fluorescence microscopy becomes a more and more important tool for the study of plant development, there is also a need for fluorescent stains to visualise non-viable cells.

The fluorescent stain SYTOX green exhibits bright fluorescence when bound to DNA, but cannot penetrate the plasma membrane of viable plant cells. These properties make the dye a useful tool for the detection of membrane permeabilisation in unicellular organisms [10,11]. In higher plants, however, SYTOX green is most commonly used to stain the nuclei of dead cells in fixed tissues [12]. We have previously used SYTOX green to identify non-viable cells in embryos of mutant plants [13]. Here we show that this dye and its spectral variants, SYTOX blue and SYTOX orange, can be easily used to identify non-viable cells within plant tissue. Moreover, the spectral properties of the SYTOX dyes allows them to be used in combination with other fluorescent stains or in plants expressing fluorescent proteins, such as the gene for GREEN FLUORESCENT PROTEIN (GFP).

## Results and Discussion

### Spectral variants of SYTOX dyes stain the nuclei in fixed plant tissue

**Figure 1:**
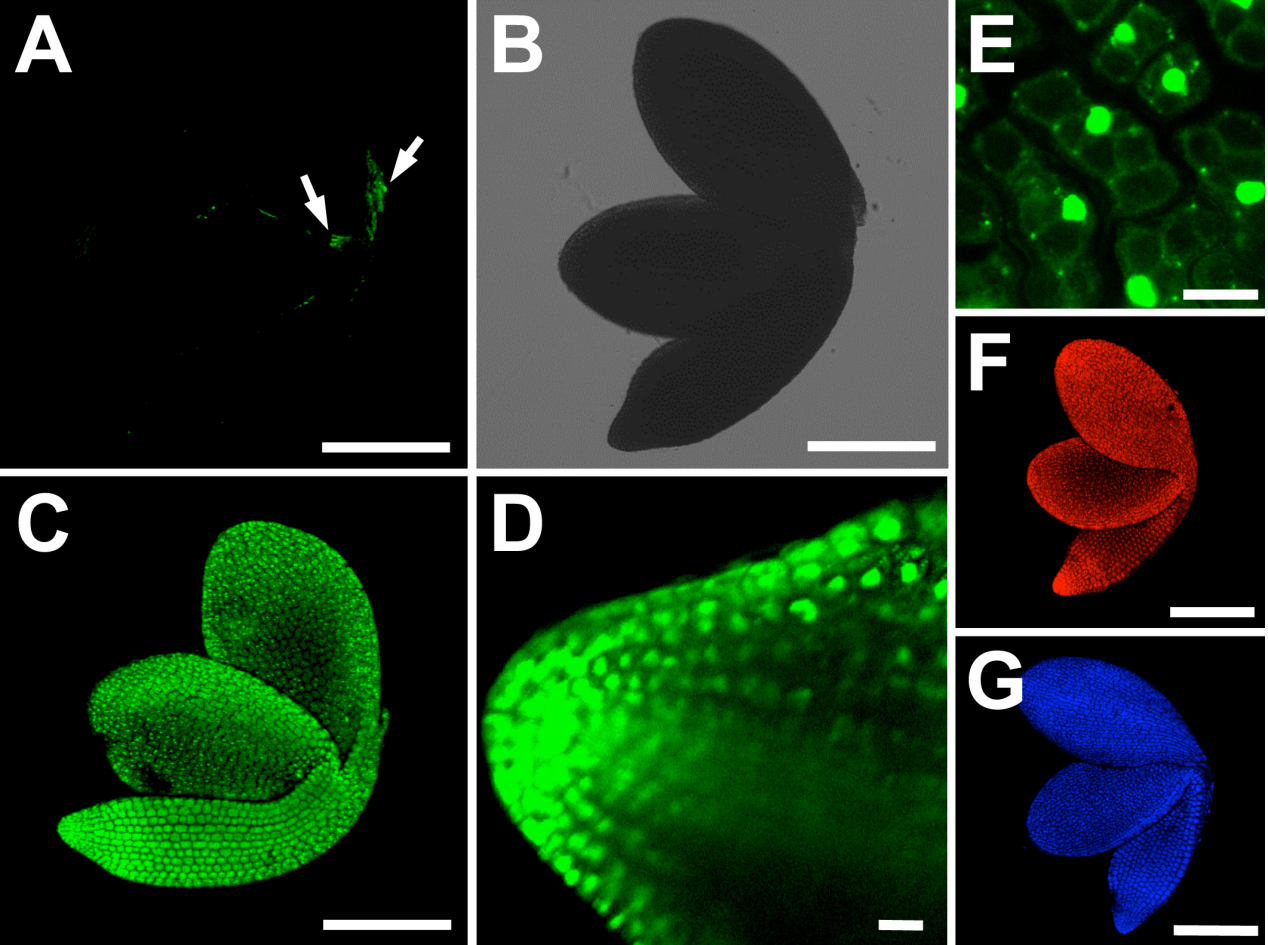
**SYTOX dye variants efficiently stain nuclei in fixed plant material**. **(A) **Live embryo stained with SYTOX green. Only a few cells are stained (fluorescence shown in green), which have been wounded during the dissection of the embryo from the seed coat (arrows). **(B) **Transmission image of the embryo shown in **(A)**. **(C) **Fixed embryo stained with SYTOX green. **(D) **CLSM section through the root of a fixed embryo stained with SYTOX green. The stain penetrates into deeper tissue layers. **(E) **Higher magnification of **(C) **showing that predominantly the nuclei are stained in the embryonic cells. **(F) **Fixed embryo stained with SYTOX orange (fluorescence shown in red). **(G) **Fixed embryo stained with SYTOX blue (fluorescence shown in blue). All images with the exception of **(D) **are overlay projections of CLSM images. Scalebars: 10 μm in **(D) **and **(E)**, otherwise 200 μm.

To assess the property of the different SYTOX dyes and their suitability for the staining of non-viable cells, embryos of *Arabidopsis thaliana *were dissected from their seed coats, fixed with 4% paraformaldehyde, and stained with SYTOX green, blue, or orange for 5 minutes. Stained embryos were easily detectable with a fluorescence microscope. Confocal laser scanning microscopy (CLSM) images were taken with the excitation and emission wavelengths indicated in Table 1. All cells of the fixed embryos were stained well with all three dyes (Figure 1C to 1G), while freshly prepared embryos only showed the occasional staining of one or two cells that had probably been damaged during preparation (Figure 1A, B). At higher magnification it became obvious that predominantly the nuclei of the fixed plant tissue were stained (Figure 1D, E). To a variable extent staining of cytoplasmic content was also observed. The stains seemed to penetrate well through the tissue layers. However, due to the scattering of laser light in deeper tissue layers, which is usually observed during live plant imaging [14], the fluorescent signal appeared weaker towards the centre of the plant tissues (Figure 1D). Taken together, the three spectral variants of SYTOX dye that we tested stained the nuclei of fixed plant cells, while cells in living tissue were not stained.

**Table 1:** Excitation and emission wavelengths (in nm) used.

	excitation maximum	laser line used	emission maximum	emission collected
SYTOX blue	444	458	480	475 to 500
SYTOX green	504	488	523	510 to 560
SYTOX orange	547	568	570	580 to 610
GFP	457	488	504	510 to 545
Propidium iodide	536	488	617	610 to 650
Fluorescein diacetate	494	488	518	505 to 525

The first and third columns show excitation and emission maxima of the fluorescent dyes and proteins used (as indicated by the suppliers). The second and fourth columns show the excitation and emission wavelengths used in our experiments.

### Non-viable cells are stained selectively

**Figure 2:**
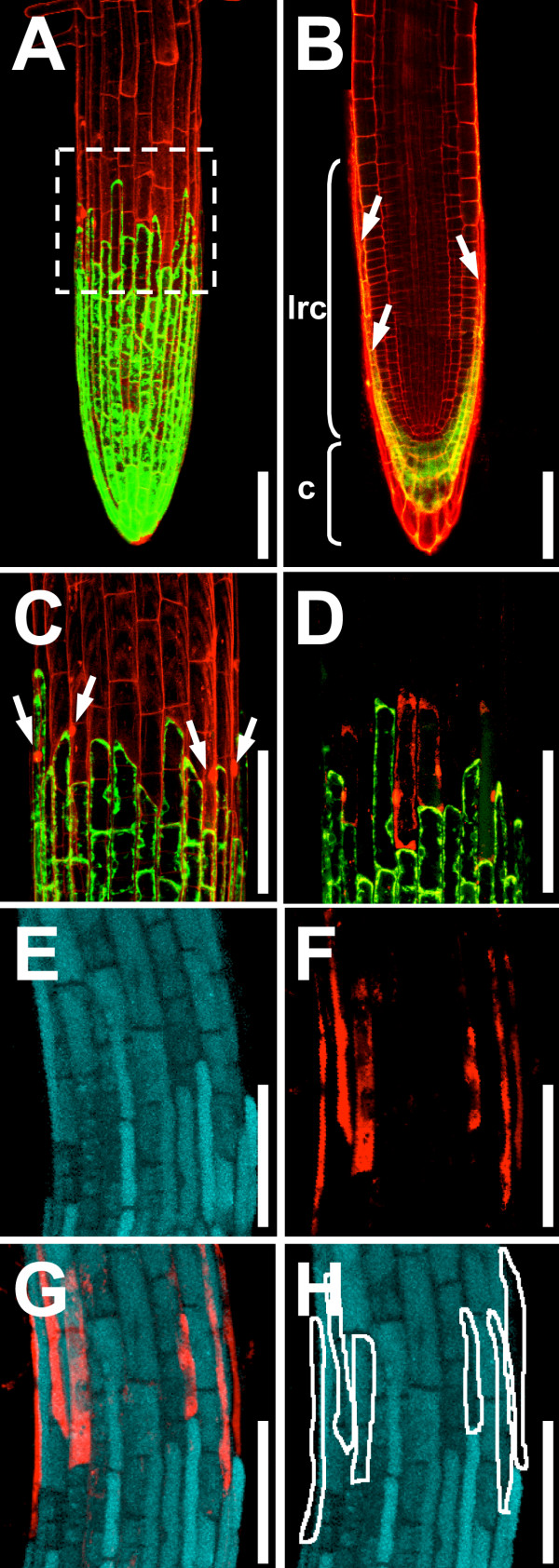
**SYTOX orange staining of non-viable lateral root cap cells**. **(A) **to **(D) **Enhancer-trap line Q0171 expressing GFP (green) in the columella (c) and lateral root cap (lrc). **(A)**, **(C) **Overlay projection image of Q0171 stained with propidium iodide. The end of the lateral root cap (box in **(A)**) is magnified in **(C)**. Propidium iodide stain (red) outlines cells and stains nuclei of non-viable root cap cells (arrows in **(C)**). **(B) **CLSM image showing a virtual optical section through the middle of a Q0171 root stained with propidium iodide. To visualise cells in the centre of the root, the root was stained with propidium iodide for an extended time (10 minutes) and therefore cells at the periphery of the root are heavily stained. The lateral root cap is marked with an arrow and a bracket shows the length of the lateral root cap. **(D) **Overlay projection image of line Q0171 stained with SYTOX orange: non-viable cells are stained selectively. **(E) **to **(H) **Overlay projection images of the end of the lateral root cap of wild type roots stained with fluorescein diacetate (labels living cells, shown in cyan) and SYTOX orange (red). **(E) **Fluorescein diacetate fluorescence. **(F) **SYTOX orange fluorescence in the same root. **(G) **Overlay of **(E) **and **(F)**. **(H) **Scheme outlining the SYTOX orange stained cells. All roots are 5 days old. Scalebars: 100 μm.

The root cap, consisting of columella and lateral root cap, encloses the *Arabidopsis *root tip. The lateral root cap terminates as meristematic cells transition into the rapid elongation zone of the root (Figure 2) [6]. Cells at the end of the lateral root cap die, thus exposing the root epidermis [6].

The enhancer-trap line Q0171 expresses *GFP *in the columella and lateral root cap cells [15]. Figure 2 shows root tips of line Q0171 stained with propidium iodide. The fluorescent dye propidium iodide is commonly used to stain plant roots (e.g. [15]). It outlines cells in living plant tissue and its emission wavelengths do not overlap with those of GFP (Table 1). Like the SYTOX dyes, propidium iodide is able to penetrate the plasma membrane of non-intact plant cells, where it binds to DNA. However, while propidium iodide shows an enhancement of only 20- to 30-fold when bound to DNA, the SYTOX dyes are 500 times more fluorescent in this situation (supplier's information). *GFP *expression in line Q0171 nicely marked the lateral root cap cells (Figure 2A to 2D) and propidium iodide stained the outlines of all root cells (Figure 2A to 2C). Moreover, some nuclei of cells immediately adjacent to the GFP expressing cells were also stained, indicating that these cells may be lateral root cap cells that have died (arrows in Figure 2C).

To see if we could label those cells specifically, we stained Q0171 roots with SYTOX orange for 5 minutes. SYTOX orange was chosen because its excitation and emission wavelengths do not overlap with those of GFP. This makes it possible to simultaneously image GFP and SYTOX orange fluorescence in the same plant. Indeed, SYTOX orange labelled only a few cells at the end of the lateral root cap (Figure 2D).

In order to have convincing proof of the specificity of the SYTOX dyes for non-viable cells, we stained wild type roots with SYTOX orange and fluorescein diacetate, a fluorescent dye that only stains the cytoplasm of living cells [16]. No overlap of fluorescein diacetate and SYTOX orange fluorescence could be detected in cells at the end of the lateral root cap (Figure 2E to 2H) [see Additional file 1].

To test if the SYTOX dyes were still specific to non-viable cells after longer staining times, we stained roots for 20 minutes with SYTOX green, blue, and orange [see Additional file 2]. In our hands SYTOX orange labelled the non-viable cells in the root most specifically. SYTOX green and blue dyes exhibited strong intracellular staining of non-viable lateral root cap cells but also weak staining around the plasma membrane of viable cells. To summarize, all tested SYTOX dyes selectively marked non-viable cells in the *Arabidopsis *root. SYTOX orange exhibited more specificity than SYTOX blue and green. Moreover, SYTOX orange can be conveniently used in plants expressing *GFP*. The different spectral SYTOX dye variants cover a broad spectrum of wavelengths (Table 1) and therefore several other combinations of SYTOX dyes with fluorescent proteins or stains will be possible.

### SYTOX dyes can be used for analysis of cell death in mutant plants

**Figure 3:**
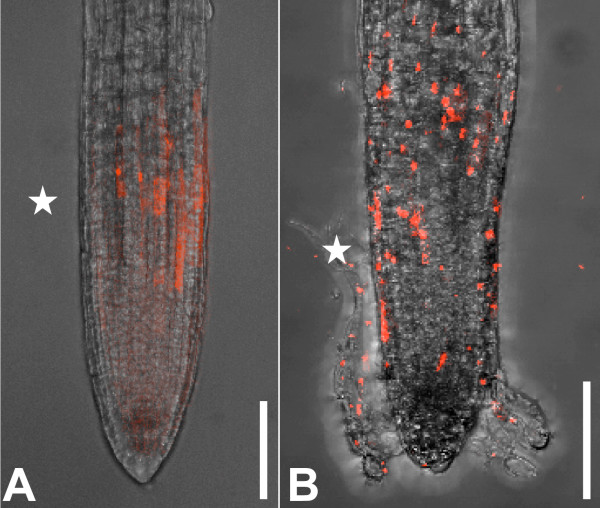
**SYTOX orange staining of *trn *mutant shows non-viable cells in the epidermis**. **(A) **5 day-old wild type root stained with SYTOX orange. Only cells at the end of the lateral root cap are dying. **(B) **7 day-old trn root. Random cells in the epidermis are stained, showing their non-viability. All images are overlay projections of CLSM images. The end of the lateral root cap is marked with an asterisk. Scalebars: 100 μm.

To test SYTOX dye utility for characterisation of cell death in mutant plants, we stained the roots of the *trn *mutant [8]. It has been assumed that random cells in the epidermis of *trn *roots adopt lateral root cap cell identity and then die in the root elongation zone. To visualise this process, we stained 7 day-old *trn *roots with SYTOX orange and observed them with CLSM. As predicted, individual SYTOX orange stained cells were observed in the outermost cell layer of the elongation zone of *trn *roots from the end of the lateral root cap to the beginning of root hair outgrowth (Figure 3B). This random cell-labelling pattern was not observed in wild type roots (Figure 3A). We conclude that the SYTOX dyes are useful to study mutant phenotypes associated with cell death.

## Conclusion

With the normal constraints of CLSM on live plant tissue we found the fluorescent SYTOX dyes, SYTOX green, blue, and orange, useful and convenient for the identification of non-viable plant cells within living plant tissue. Stain penetration into plant tissue was good, and non-viable cells fluoresced brightly and could be easily identified and imaged. A combination of the stains with plants expressing fluorescent proteins was possible, as emission and excitation spectra did not overlap. The staining assay is fast and simple, allowing for the design of rapid screens during mutant analysis or during the study of plant development using fluorescence microscopy or CLSM. Therefore the SYTOX dyes will be a useful tool for the study of plant development, physiology, and/or environmental responses.

## Methods

### Transgenic lines

The GAL4-GFP enhancer trap line [17] Q0171 is available from the Nottingham *Arabidopsis *Stock Centre (NASC) as stock number N9207. The trn1-1 line was a gift from Liam Dolan, John Innes Centre, Norwich.

### Growth conditions

Plants were germinated and grown under a 16 h light, 8 h dark photoperiod on media containing 0.5× Murashige and Skoog salt mixture (MS), 0.5 g/l 2-(N-morpholino) ethanesulfonic acid (MES) pH 5.7 and 0.7% agar.

### Staining procedures

For staining of embryos, seeds were imbibed over night in water before being dissected out of their seed coats with fine needles. Fixation of tissue was performed over night at 4°C in a solution of 4% paraformaldehyde in 100 mM sodium phosphate buffer pH7. Propidium iodide staining: propidium iodide (Molecular Probes, Eugene, USA) was used as a 10 μg/ml solution in water. Plants were stained for 5 minutes. SYTOX staining: SYTOX green, orange, and blue were supplied as solution in DMSO (Molecular Probes, Eugene, USA) and were diluted to 250 nM in water. Staining times are indicated in the text. Fluorescein diacetate staining: Fluorescein diacetate (Sigma-Aldrich, Saint Louis, USA) was used as a 5 μg/ml solution in water. Plants were stained for 20 to 30 minutes. All images were taken within the first 30 minutes after staining.

### Confocal laser-scanning microscopy (CLSM)

All plant material was imaged in water on a glass slide and covered with a cover slip. CLSM was performed using a Leica TCS NT/SP microscope. Excitation and collection wavelengths are summarized in Table 1. In most cases overlay projections were produced, i.e. several images were taken at different focal planes and overlays were produced with the confocal microscope software.

## Competing interests

The authors declare that they have no competing interests.

## Authors' contributions

ET designed, coordinated and carried out the experiments and drafted the manuscript, JH participated in the coordination of the experiments. All authors read and approved the final manuscript.

## Supplementary Material

Additional file 1 **Additional examples for the specificity of SYTOX dyes for non-viable cells**. Shown are roots stained with fluorescein diacetate (cyan) and SYTOX orange (red). **(A), (B) **Detail of root showing cells at the end of the lateral root cap. (A) Fluorescein diacetate fluorescence. **(B) **Overlay of fluorescein diacetate and SYTOX orange fluorescence. While a SYTOX orange stained cell is seen in **(B)**, a gap can be seen in **(A) **at the same position. **(C)**. Confocal optical section through a root showing long cells at the end of the lateral root cap being selectively stained with SYTOX orange. Scalebars: 50 μm. Click here for file

Additional file 2 **Specificity of SYTOX dyes after longer staining times**. Shown are roots stained for 20 min with SYTOX green **(A)**, blue **(B)**, or orange **(C)**. All dyes stain most brightly the non-viable cells at the end of the lateral root cap. SYTOX green and SYTOX blue show slight background staining in the rest of the root. All images are overlay projections of CLSM images. Scalebars: 100 μm. Click here for file
