# Supplementary figures and images for: A simple way to identify non-viable cells within living plant tissue using confocal microscopy
Source: Plant Methods. 2008 Jun 23;4:15. doi: 10.1186/1746-4811-4-15 (PMC2442066; doi:10.1186/1746-4811-4-15)

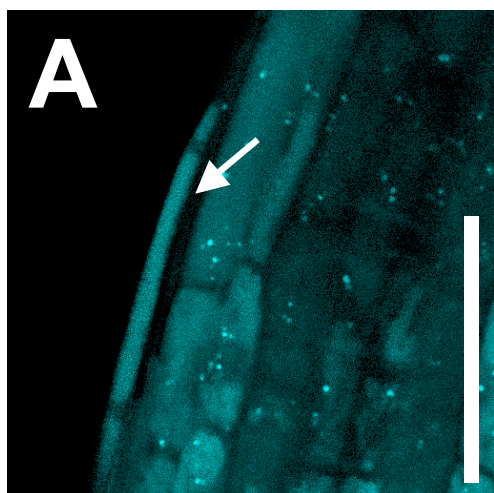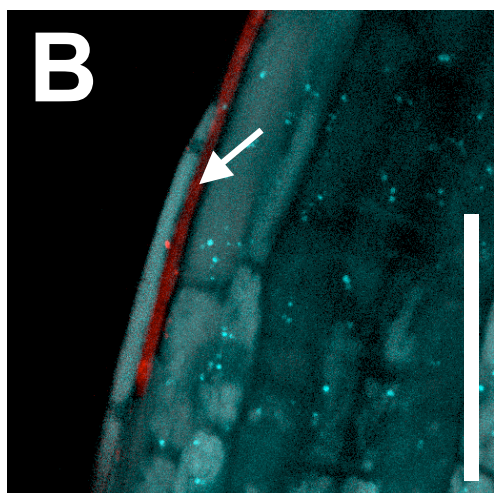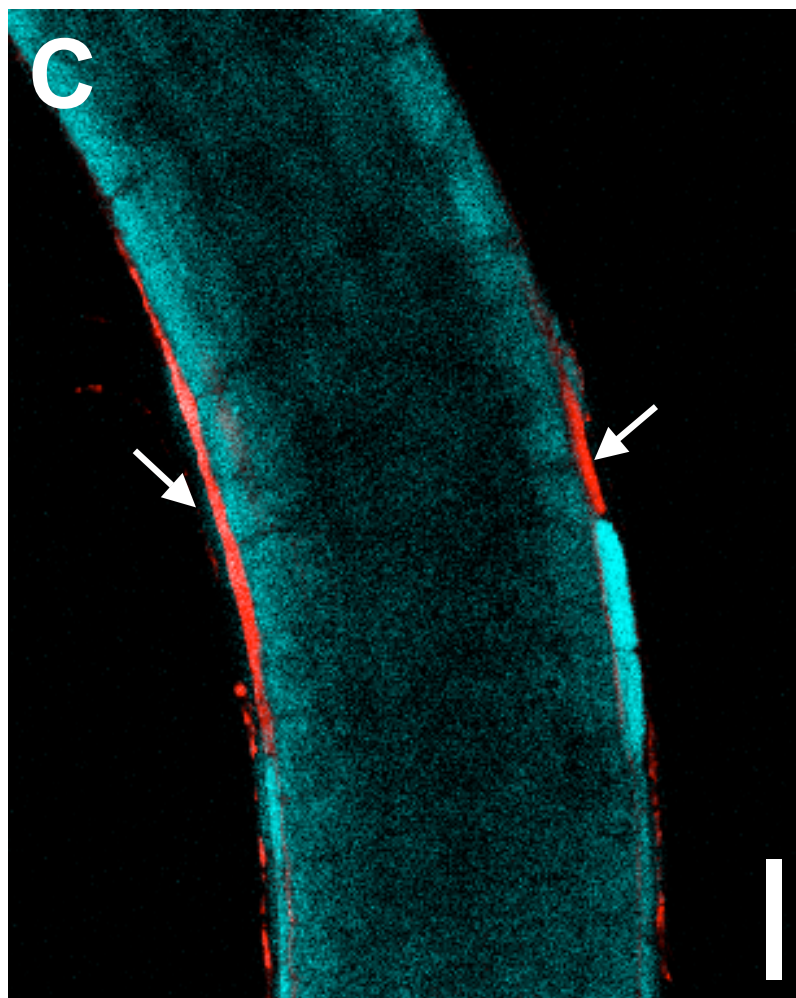

Supplement: Additional file 1 — Additional examples for the specificity of SYTOX dyes for non-viable cells. Shown are roots stained with fluorescein diacetate (cyan) and SYTOX orange (red). (A), (B) Detail of root showing cells at the end of the lateral root cap. (A) Fluorescein diacetate fluorescence. (B) Overlay of fluorescein diacetate and SYTOX orange fluorescence. While a SYTOX orange stained cell is seen in (B), a gap can be seen in (A) at the same position. (C). Confocal optical section through a root showing long cells at the end of the lateral root cap being selectively stained with SYTOX orange. Scalebars: 50 μm. [file 1746-4811-4-15-S1.pdf]

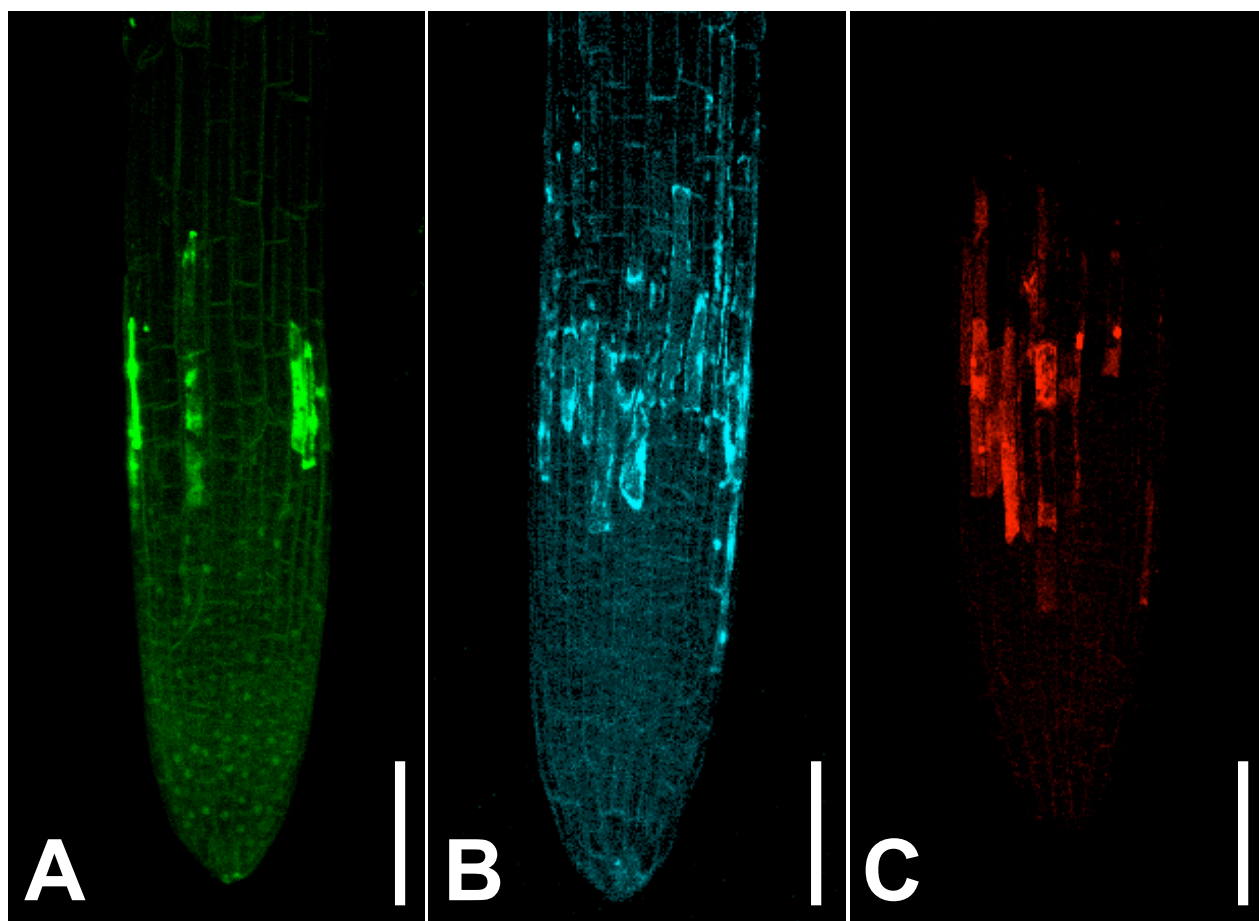

Supplement: Additional file 2 — Specificity of SYTOX dyes after longer staining times. Shown are roots stained for 20 min with SYTOX green (A), blue (B), or orange (C). All dyes stain most brightly the non-viable cells at the end of the lateral root cap. SYTOX green and SYTOX blue show slight background staining in the rest of the root. All images are overlay projections of CLSM images. Scalebars: 100 μm. [file 1746-4811-4-15-S2.pdf]
